# Editorial: Physical Activity, Health Equity and Health Related Outcomes

**DOI:** 10.3389/fpubh.2022.828108

**Published:** 2022-02-24

**Authors:** Noël C. Barengo, Ahmad Alkhatib

**Affiliations:** ^1^Department of Translational Medicine, Herbert Wertheim College of Medicine, Florida International University, Miami, FL, United States; ^2^Department of Public Health, Faculty of Medicine, University of Helsinki, Helsinki, Finland; ^3^Department of Health Policy and Management, Robert Stempel College of Public Health and Social Work, Florida International University, Miami, FL, United States; ^4^School of Health and Life Sciences, Teesside University, Middlesbrough, United Kingdom

**Keywords:** health equity (MeSH), sedentarism, urban–rural, diabetes risk, environment

It is now established that physical activity (PA) decreases the risk of developing long term non-communicable chronic diseases such as cardiovascular disease, cancer, and diabetes (1). Low PA levels combined with increased risks of major causes of mortality in most countries has led to characterizing physical inactivity as a global pandemic (2). Disability-adjusted life years due to low PA levels have almost doubled from 8.61 million in 1990 to 15.7 million in 2019 (2). Understanding determinants of sedentary lifestyle behavior and physical inactivity health risks and implications across different populations remains one of the biggest public health challenges (3). PA behavior is socially patterned with lower participation rates among women, different racial/ethnic groups, people with less education, worse access to health care and health insurance, people with physical, mental, and cognitive disabilities or older adults.

The American College of Sports Medicine (ACSM) has developed a national roadmap that supports achieving health equity through a physically active lifestyle (4). The actionable, integrated pathways that provide the foundation of ACSM's roadmap include the following: (1) communication-raising awareness of the issue and magnitude of health inequities and conveying the power of PA in promoting health equity; (2) education-developing resources to improve cultural competency for health care providers and fitness professionals as well as developing new community-based programs for lay health workers; (3) collaboration-building partnerships and programs that integrate existing infrastructures and leverage institutional knowledge, reach, and voices of public, private, and community organizations; and (4) evaluation-ensuring that ACSM attains measurable progress in reducing PA disparities to promote health equity.

This special issue of Frontiers in Public Health “*Physical Activity, Health Equity and Health Related Outcomes*” produced topical research conducted within different populations (Armenia, China, Colombia, the UK, and the US). Wide range of analyses included PA and sedentary behaviors prevalence, associations and mapping of chronic disease risks such as obesity and diabetes risks in cities and community groups, disparity in PA access and built environment and various socio-economic and demographic statuses determinants. Three studies analyzed health inequities and PA during the Covid-19 pandemic.

Tcymbal et al. analyzed current levels of physical inactivity and sedentary behaviors among the adult population of Armenia. Their data revealed that sedentary behavior was more common among men, students, people who were retired, unemployed, residents of Yerevan, and adults aged under 30 and over 45 years. A study conducted in the UK population (Rogers et al.) analyzed whether lockdown had a disproportionate impact on PA behavior in groups who were, or who perceived themselves to be, at heightened risk from COVID-19. Finally, Zhou et al. investigated the changes in PA and sedentary time among Chinese youths at different stages after the COVID-19 outbreak. They revealed that COVID-19 had both immediate and longer-term impacts on self-reported PA and sedentary behaviors among Chinese youths. These studies concluded that policies on maintaining or improving PA intensity during lockdowns should consider vulnerable groups of adults including those with chronic diseases or self-perceptions of being at risk and the importance of access to green or open spaces in which to exercise. Relevant efforts should alsoget youths physically moving again.

Two studies in the Special Topic collection analyzed the associations between built environment, neighborhood walkability and access to free or low-cost facilities and PA in the US population. Budd et al. studied the associations among overall perceived neighborhood walkability, racial discrimination stress, and having a chronic health condition. They also evaluated whether overall perceived neighborhood walkability moderates the association between racial discrimination stress and having a chronic health condition. Their data found that the overall perceived neighborhood walkability was inversely associated with racial discrimination stress, but not associated with having a chronic health condition. They concluded that perceived neighborhood crime safety, but not infrastructure or aesthetics, matters when it comes to the link between racial discrimination stress and having a chronic health condition among Hispanics/Latinos. Moreover, another research in the US population assessed whether access to free or low-cost recreational facilities was associated with meeting the AHA PA guidelines (Andrade et al.). Their results revealed that having access to free or low-cost recreational facilities such as parks, walking trails, bike paths and courts was associated with meeting the American Heart Association PA guidelines. Thus, increasing prevalence and awareness of neighborhood recreational facilities could assist in access to these facilities and increase the ability of individuals to meet PA guidelines.

Two studies were conducted in the Colombian population. Panciera-di-Zoppola et al. analyzed PA levels among ethnic groups in La Guajira, Colombia based on ethnic and sociodemographic factors. They revealed that participants of ≥47 years of age, and those with only a primary education presented a lower probability of complying with PA recommendations, while those who lived in large municipalities (Riohacha) displayed a larger probability of compliance. Furthermore, their data showed that Indigenous and Afro-Colombian people in a low social class were more likely to comply with PA recommendations, while residing in a smaller municipality (Manaure) was associated with a lower PA compliance. On the other hand, Acosta et al. demonstrated that low levels or PA and sedentary behaviors are prevalent among a large proportion of populations with high risk of type 2 diabetes living in Bogota and Barranquilla, Colombia. Their analysis in these two large cities recommends urgent PA interventions among high-risk populations to reduce type 2 diabetes risk.

Finally, two studies conducted in the Chinese population analyzed health equity among rural residents with various socio-economic and demographic statuses in Yunnan Province (Li et al.) and the joint associations of socioeconomic status or PA on obesity measures (Pan et al.) in an urban setting. Their studies suggested that more attention should be paid to females, the aged, ethnic minorities, farmers, the divorced or widowed, residents with low income and low educational level, and those with chronic diseases. Also, women were more susceptible to obesity concerning low socioeconomic status and that adequate PA may be a potential target for mitigating the negative effect of low socioeconomic status on obesity in women. Ensuring equity and equality has also been analyzed in terms of receiving medical care and drug welfare in disadvantaged communities in Chinese patients with chronic diseases in Gansu, Sichuan, Hebei, and Zhejiang using the Bivariate Theil-T Index method (Tang et al.). Understanding PA determinants in the context of health equity and health related outcomes is key to developing targeted successful public health interventions which ultimately reduce disease risks across wide range of populations.

## Author Contributions

All authors listed have made a substantial, direct, and intellectual contribution to the work and approved it for publication.

## Conflict of Interest

The authors declare that the research was conducted in the absence of any commercial or financial relationships that could be construed as a potential conflict of interest.

## Publisher's Note

All claims expressed in this article are solely those of the authors and do not necessarily represent those of their affiliated organizations, or those of the publisher, the editors and the reviewers. Any product that may be evaluated in this article, or claim that may be made by its manufacturer, is not guaranteed or endorsed by the publisher.
